# Plasma Nickel Levels Correlate with Low Muscular Strength and Renal Function Parameters in Patients with Prostate Cancer

**DOI:** 10.3390/diseases10030039

**Published:** 2022-06-30

**Authors:** Antoni Alegre-Martínez, María Isabel Martínez-Martínez, José Rubio-Briones, Omar Cauli

**Affiliations:** 1 Frailty and Cognitive Impairment Organized Group (FROG), University of Valencia, 46010 Valencia, Spain; antoni.alegre@uchceu.es (A.A.-M.); m.isabel.martinez@uv.es (M.I.M.-M.); 2 Department of Biomedical Sciences, Cardenal Herrera University CEU, Avenida Seminario, s/n, Moncada, 46113 Valencia, Spain; 3 Department of Nursing, University of Valencia, 46010 Valencia, Spain; 4 Department of Urology, Fundación Instituto Valenciano de Oncologia, 46009 Valencia, Spain; jrubio@clinicadoctorrubio.es

**Keywords:** prostate cancer, endocrine effects, metal, environment, estrogens, androgens

## Abstract

Nickel is associated with cancer in occupational exposure. However, few studies have been devoted to analyzing the effects of nickel at environmental concentrations in cancer patients. In this work, the concentration of nickel in blood samples from patients with prostate cancer (PCa) was evaluated because this metal displays androgenic and estrogenic effects that play a crucial role in prostate carcinogenesis and treatment. We, therefore, compared blood nickel concentration in patients with PCa (non-occupationally exposed) (*n* = 46) with those in control age-matched individuals (*n* = 46). We also analyzed if there was any association between sociodemographic factors, clinical variables, geriatric evaluation assessment results, blood cell counts, or biochemical, androgen and estrogen concentrations. Using inductively coupled plasma-mass spectroscopy on the plasma samples, we observed a mean nickel level of 4.97 ± 1.20 µg/L in the PCa group and 3.59 ± 0.49 µg/L in the control group, with a non-significant effect (*p* = 0.293) between the two groups. The nickel concentration was significantly correlated with patient age (*p* = 0.005) and reduced handgrip strength (*p* = 0.003). Regarding biochemical parameters, significant associations were found with the renal glomerular filtration rate (*p* = 0.024) and blood urea levels (*p* = 0.016). No significant correlations were observed with other blood analytical parameters or testosterone or estradiol levels. These specific renal function and muscle strength effects were observed at environmental nickel exposure levels believed to be safe or at least far from the high concentrations observed after occupational exposure. Therefore, these parameters deserve further study, given that they could help pinpoint further public health concerns regarding nickel exposure in the general population.

## 1. Introduction

In 1996, the World Health Organization (WHO) classified nickel as a potentially essential element for human health, claiming that certain pathological signs such as poor growth or depressed hematopoiesis could be attributable to severe nickel (Ni) deficiency [1]. Nonetheless, inhalation exposure to Ni in occupational settings is one of the major routes of Ni-induced toxicity in the respiratory tract, lung tissue, and immune system [2]. Inhalation exposure in non-work environments can also affect the general population. However, exposure to Ni in the general population usually occurs by oral ingestion through water and food, given that Ni is also a contaminant present in drinking water and several foodstuffs [3,4,5,6] and is released during cooking [7].

Ni exposure in occupational settings has been associated with an increased risk of prostate cancer (PCa), lung and nasal mucosa cancers [2,8,9,10,11,12], and breast cancer [13]. Indeed, research performed on experimental animals showed that Ni administration could induce carcinogenesis in multiple organs [14]. In Western countries, PCa is a major health problem because of its high incidence and significant mortality rates [15]. Thus, primary prevention not only reduces the significant economic burden of diagnosis and treatment but can also lessen the enormous emotional distress of patients and their families and loved ones. To this end, it is imperative for researchers to increase their knowledge of the environmental risk factors involved in Ni etiopathogenesis. This will allow them to tailor preventive strategies and more directly surveil individuals with known risk factors for PCa.

Sorahan et al. reported a significantly increased risk of PCa mortality with relatively high occupational Ni exposure [8]. Indeed, several studies have found increased concentrations of Ni in different cancerous tissues. For example, Millos et al. observed that the Ni concentrations in breast cancer tissues exceeded that of non-cancerous or adjacent tissues by a factor of more than three [16]. Similarly, Yaman et al. determined trace metal concentrations in cancerous stomach tissues and found an increased Ni content compared to non-cancerous tissue [17].

In contrast, the Ni concentration in PCa tissue has been reported as being lower [18] or higher [19,20] compared to benign prostate tissue. The levels of Ni present in blood samples from patients with PCa have also been reported as higher compared to controls [21]. The mechanisms by which Ni promotes cancer growth are diverse and include the induction of DNA alterations, inhibition of intercellular transmission mechanisms, formation of DNA–protein crosslinks, inhibition of the maintenance of nucleotide excision, oxidative stress, and DNA methylation [22,23,24,25,26,27,28,29,30,31,32].

A role for androgens in prostate tumor progression is already well recognized, while estrogens may also cooperate with androgens in prostate carcinogenesis [33]. Indeed, some metals have estrogenic and/or androgenic activities and may increase the PCa risk through this mechanism [33]. Of note, epidemiological data indicate associations between exposure to environmental endocrine disruptors and adverse health outcomes in prostate disease in adult males [34,35,36]. In the case of Ni, it has been reported this metal can bind and activate estrogen receptors and can contribute to the development of breast cancer and PCa [37]. In addition, Ni^2+^ can replace Zn^2+^ in the DNA-binding affinity of estrogen alpha receptors [38,39]. In this sense, Ni-induced estrogenic activity can synergize with DNA alterations induced by Ni to promote cancer growth.

Given all the above, the main objectives of this current study were to (1) replicate the previously found difference in Ni blood concentrations in a cohort of Spanish men with PCa compared with aged-matched controls; (2) evaluate the association between the levels of Ni and estrogen and androgen in blood; (3) assess any association between Ni concentration and sociodemographic, clinical factors, or inflammatory markers in PCa patients.

## 2. Material and Methods

### 2.1. Study Population

A cross-sectional clinical trial was carried out in patients with PCa (*n* = 46) and followed up by the Department of Urology at an oncological center (Urology Oncology Department, Fundacion IVO, Valencia, Spain). The inclusion criterion was a diagnosis of PCa (any stage). To compare the levels of Ni burden in these patients with those in men without PCa (or any other cancer type), we measured Ni in blood samples of a control group of age-matched men (*n* = 46) living in nursing homes in the Valencia province.

The exclusion criteria for both groups were severe cognitive impairment (Mini-Mental State Examination [MMSE] score < 21) or a severe psychiatric disorder. The trial was carried out in compliance with the guidelines set out in the Declaration of Helsinki, and the study protocol was approved by the local Ethics Committee (University of Valencia, Reference number: H1511682610849). All the participants gave their written informed consent before being enrolled in the study.

### 2.2. Sociodemographic Factors and Geriatric Evaluation

The variables studied included sociodemographic characteristics (age, marital status, body mass index [BMI], and smoking status) and clinical PCa variables (clinical stage at diagnosis, time since the diagnosis, and prior prostatectomy). We also evaluated the presence of associations between nickel concentration and psychological and functional parameters since Ni exposure has been associated with these parameters in exposed individuals [40,41,42].

The Spanish version of the geriatric functional status assessment, which uses the Barthel index, was employed to define the ability of participants to perform the basic activities of daily living [43]. This test measured the level of patient independence for each of the following 10 items: feeding, bathing, dressing and undressing, grooming, bowel control, bladder control, getting on and off a toilet, transfers (e.g., from an armchair to a bed), walking on a level surface (or propelling a wheelchair if unable to walk), and ascending and descending stairs. The index has a score range of 0–100, where 0 is total dependence, and 100 corresponds to total independence.

The Spanish version of the MMSE test was used to detect cognitive impairment. It evaluates different items, grouped into five sections: orientation to time and place, immediate memory, attention and calculation, delayed (evocation) recall, and language and visual construction. It has a score range of 0–30, with the highest scores indicating better performance [44]. The International Physical Activity Questionnaire (IPAQ), validated in Spanish, was used to assess physical activity as a function of time and intensity when measuring low physical activity levels [45].

Sleep quality was assessed using the Athens Insomnia Scale (AIS), also validated in the Spanish language [46], comprising eight items: sleep induction, awakenings during the night, final awakening, total sleep duration, sleep quality at night, well-being during the day, functioning capacity during the day, and sleepiness during the day. Each item on the AIS can be rated 0–3, with 0 corresponding to ‘no problem at all’ and 3 a ‘very serious problem’. The scale has a score range of 0–24, where 0 denotes the absence of any sleep-related problems and 24 corresponds to the most severe degree of insomnia.

Muscle weakness was measured according to handgrip strength (in Kg), taking the palmar grip strength obtained with a hydraulic dynamometer (Jaymar, J.A. Preston, Corp., Jackson, MS, USA) as a benchmark. Three consecutive measurements were performed in each hand, alternating the arms and leaving a muscle recovery time of approximately one minute between trials. The highest value from the three measurements was used in further evaluations in accordance with the standards for Hispanic Established Populations in Epidemiological Studies of the Elderly [47].

### 2.3. Nickel Determination

Ni content was analyzed in plasma samples using inductively coupled plasma-mass spectroscopy (ICP-MS) on an Agilent 7900 machine. A 100-µL aliquot of each plasma sample was placed into a beaker and then wet-digested in duplicate with 65% nitric acid (Suprapure, Merck, Darmstadt, Germany) and analytical grade 60% perchloric acid (Ultrapure, Merck; 3:1). In order to verify the analytical results obtained, an analytical quality control program was applied. Several analytical blanks (prepared exactly according to the same procedure applied to the plasma samples) were included in all batches. The corresponding results were used to calculate the limit of detection (LOD) for each of the elements considered (as 3 times the standard deviation of the blank divided by the slope of the calibration curve). The LOD values obtained were low enough to enable the determination of all elements considered. The recovery efficiencies for Ni were measured by adding a standard solution to the samples. Ni recovery rates in blood were 111.1–115.9%, and the method detection limits (MDLs) were 1.00 μg/L. The commercially available Certified Reference Materials were used as quality controls (QCs) in the ICP-MS analysis of blood and plasma samples after reconstituting as per the manufacturer’s instructions: UTAK Trace Elements Serum Control Normal Range (UTK1) and UTAK Trace Elements Serum Control High Range (UTK2) (PM Separations). Two sets of quality controls of UTK1 and UTK2 for plasma samples were determined at the beginning of the run and end of each run of 20 samples. The laboratory of the University of Valencia had an ISO9001 certification for the provision of analytical services.

### 2.4. Measurement of Hematological and Biochemical Markers

Blood serum (5 mL) was obtained by collecting blood in Becton Dickinson (BD) Vacutainer tubes (Becton Dickinson, Franklin Lakes, NJ, USA) and centrifuging them at 500 g for 10 min at room temperature. All the samples were kept at 4 °C to 6 °C and processed within 2 h of collection. Blood samples were obtained from each participant between 7:30 a.m and 10:00 a.m under fasting conditions of at least 8 h. Blood was obtained by collecting 10 mL of blood into each of 2 BD Vacutainer tubes containing ethylenediaminetetraacetic acid sodium salt (EDTA). After extraction, the blood samples were allowed to stand for 15 min and were then centrifuged at 1500 rpm for 10 min at room temperature. Subsequently, the plasma supernatants were aliquoted and stored at −20 °C until analysis. After thawing, the samples were centrifuged again at 1500 rpm for 10 min at room temperature to completely remove all the cells.

For all other analytical determinations, residential center control blood extractions were used. Hematological parameters (white blood cells, hemoglobin, erythrocytes, and platelets) and biochemical parameters (glucose, urea, urate, cholesterol, triglycerides, creatinine, glutamic oxaloacetic transaminase [GOT], serum glutamic pyruvic transaminase [GPT], and C-reactive protein) were measured in clinical laboratories pertaining to local public health centers. These hematological analyses included the total red blood cell count (RBC) and white blood cell count (WBC) obtained using a hemocytometer methodology (Autocrit Ultra3 Centrifuge, Becton Dickinson). We also measured testosterone and PSA as they are commonly used blood biomarkers in PCA management [48,49].

Serum analytical values were determined on a laboratory chemistry analyzer (Dimension Xpand Plus Integrated Chemistry System, Siemens, Erlangen, Germany). The plasma concentration of the inflammatory markers TNF-a and IL-6 were measured using commercial enzyme-linked immunosorbent assay kits according to the manufacturer’s instructions (TNF-a [ab100654] and IL-6 [ab46042], Abcam^®^, Cambridge, UK). To minimize assay variance, all the measurements were conducted in duplicate on the same day.

### 2.5. Statistical Analysis

Descriptive statistics, including a measurement of central tendency (mean), standard error of the mean, and value ranges, were used to describe all the quantitative variables. The normal distribution of each variable was assessed with the Shapiro–Wilk test to determine whether parametric or nonparametric tests should be applied. The differences between the two groups were analyzed with nonparametric Mann–Whitney U tests or parametric Student’s t-tests. The differences between the three groups were analyzed using the nonparametric Kruskal–Wallis tests or parametric analysis of variance (ANOVA), followed by posthoc testing as appropriate. The correlations between two quantitative variables were evaluated with nonparametric Spearman tests or parametric Pearson tests. A multiple linear regression model was performed to identify factors associated with plasma nickel concentration. Only variables that differed with *p* < 0.10 in the correlation analysis were selected for multivariate linear regression analysis. All models were adjusted for known variables affecting renal function parameters and muscular strength as the cancer stage, arterial hypertension, number of years passed since PCa diagnosis, body mass index and abdominal perimeter, plasma PSA, and testosterone concentration. The *p* values of <0.05 were considered statistically significant in the final multivariate model. A logistic regression analysis was used to try to make a predictive model in order to determine associations between nickel concentration categorized in tertiles with the variables identified in bivariate analyses by controlling for other potential confounding variables (cancer stage, arterial hypertension, number of years passed since PCa diagnosis, body mass index, and abdominal perimeter). SPSS software (version 25.0, IBM Corp., Armonk, NY, USA) was used for all these analyses.

## 3. Results

### 3.1. Sociodemographic Characteristics and Clinical Variables

The average participant ages in the 2 groups were not significantly different at 72.24 years in the PCa group and 74.63 years in the control group. In addition, the number of smokers was also remarkably similar between the groups (4 and 3 smokers in the PCa and control group, respectively). None of the patients at the time of blood sampling was receiving any pharmacological treatment with an anti-androgen drug or any chemotherapy drug. There were no differences between the groups in terms of educational level (Pearson chi-squared = 3.636; *p* = 0.304) or employment status (Pearson chi-squared = 3.175; *p* = 0.204), but there were some significant differences in marital status with 40 of the 46 participants with PCa being married while there were only 2 widowers in the PCa group compared to 29 in the control group (Pearson chi-squared = 52.627; *p* = 0.0001; Table 1). None of the individuals in either the PCa or control groups were occupationally exposed to Ni, and none of them had a known allergy to Ni.

### 3.2. Nickel Concentration and Its Association with Sociodemographic and Clinical Variables

**Figure 1:**
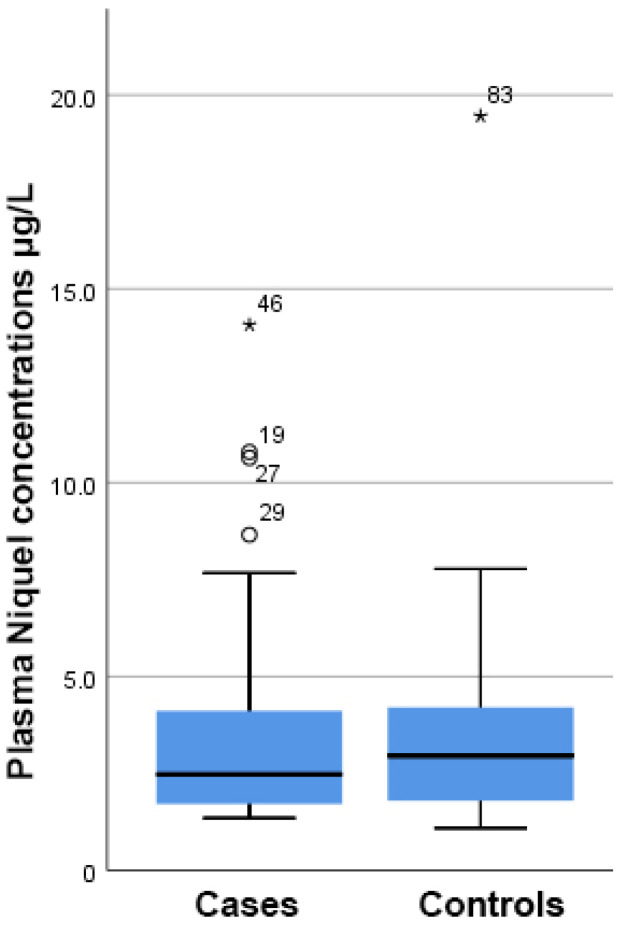
Boxplot showing that there were no differences in the plasma nickel concentration between cases and controls. On the boxplot shown here outliers are identified (“out” values (small circle) and “far out” and “Extreme values” (marked with a star)). Three extreme outliers were removed to improve the fitting.

As shown in Figure 1, there was a higher but still insignificant concentration of Ni present in the plasma samples from PCa patients compared to the control group (4.97 ± 1.20 µg/L vs. 3.59 ± 0.49 µg/L, respectively, *p* = 0.293).

**Figure 2:**
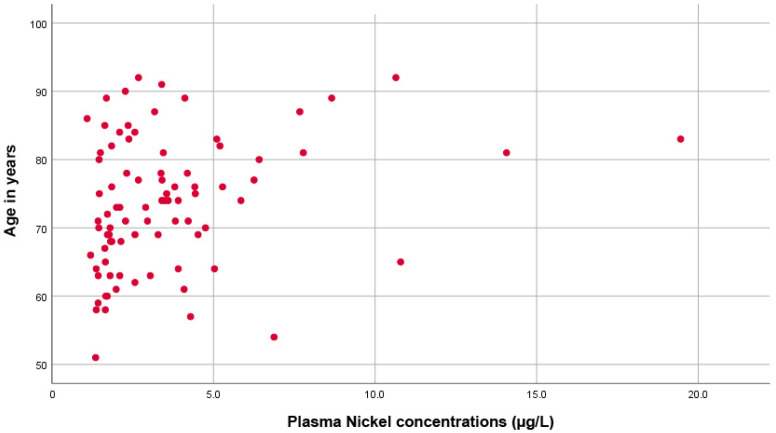
Scatter plot showing the direct proportional correlation between age and plasma nickel levels.

Moreover, as shown in Figure 2, there was a significant direct correlation between plasma Ni concentration and age (rho = 0.305, *p* = 0.005).

The correlation was even more significant in the group of PCa patients (*p* = 0.001, rho = 0.483) compared to the control group (*p* = 0.811, rho = 0.040). Kruskal–Wallis tests found no significant differences between Ni levels and educational levels (*p* = 0.101), employment status (*p* = 0.488), or marital status (*p* = 0.065). No significant changes were observed in the plasma Ni concentration between men with PCa who had or had not undergone a prostatectomy (*p* = 0.739). Multiple linear regression analysis showed that increased nickel concentration in plasma in an adjusted model taking into account the factors affecting the relationship between nickel and variables significantly associated in bivariate analyses showed significant effects with R Squared = 0.565; Adjusted R Squared = 0.347, mean square 10.951 F = 2.597, *p* = 0.041) being plasma urea concentration and age significantly (*p* < 0.05) associated. In logistic regression analysis, by selecting as the dependent variable the dichotomous variable, e.g., low plasma nickel concentration (first and second tertiles pooled together) and high nickel concentration (tertile 3), it was found to have a significant effect on urea concentration (Higher nickel concentration significantly associated with higher urea concentration, *p* = 0.03), and for age (*p* = 0.005). Glomerular filtration rate did not reach a statistical difference in the regression model (*p* = 0.091. The multivariate analysis led to a Cox and Snell R square value = 0.298 and a Nagelkerke R square value = 0.407. However, when dichotomized patients with reduced (<90 mL/min) or normal (≥90 mL/min) glomerular filtration rate, we found a significantly increased nickel concentration in patients with reduced compared with normal glomerular filtration rate (*p* = 0.01).

**Figure 3:**
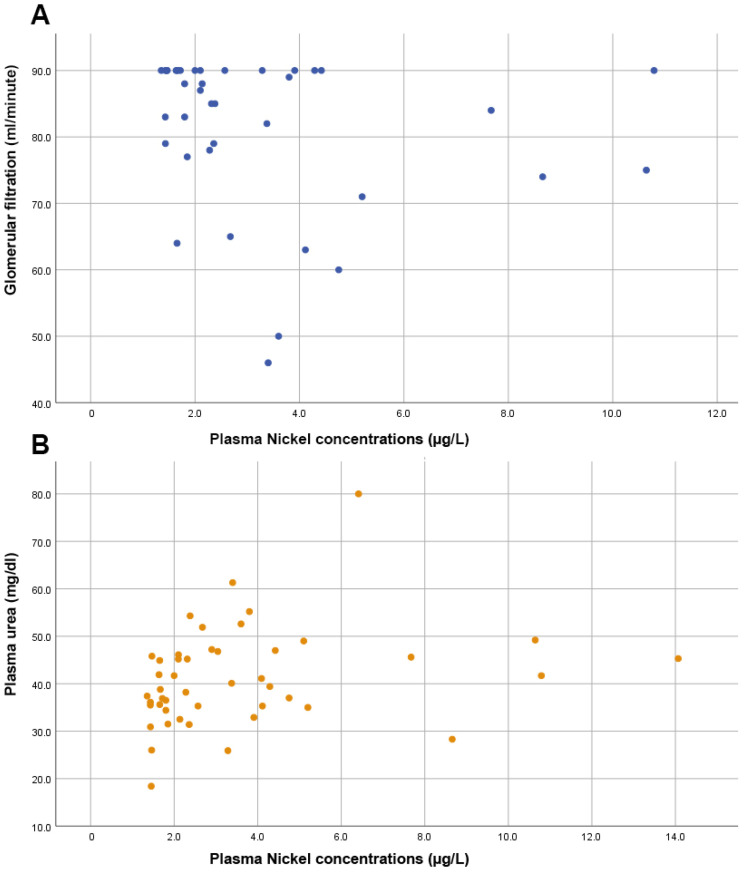
Scatter plots showing (**A**) an inversely proportional correlation between glomerular filtration and nickel levels; (**B**) the direct proportional correlation between plasma urea levels and plasma nickel levels.

Furthermore, no significant correlation was observed between Ni levels and the prostate biomarker, PSA (rho = 0.103, *p* = 0.494). As shown in Figure 3, we also found significant directly proportional correlations between plasma Ni levels and markers of renal function such as the glomerular filtration rate (rho = −0.356, *p* = 0.024) and urea levels (rho = 0.354, *p* = 0.016).

Conversely, there was no significant correlation between Ni levels and the remaining clinical variables. There were no significant coefficients or *p*-values found for correlations between plasma Ni levels and systolic blood pressure (rho = −0.103, *p* = 0.498), diastolic blood pressure (rho = 0.194, *p* = 0.196), abdominal circumference (rho = −0.144, *p* = 0.340), with other analytical parameters including leucocytes (rho = −0.152, *p* = 0.312), red blood cells (rho = −0.140, *p* = 0.361), hemoglobin (rho = −0.122, *p =* 0.418), hematocrit (rho = −0.099, *p =* 0.513), mean corpuscular volume (rho = 0.272, *p =* 0.070), mean corpuscular hemoglobin (rho = 0.134, *p =* 0.379), red cell blood distribution width (rho = 0.029, *p =* 0.851), platelets (rho = −0.109, *p =* 0.472), mean platelet volume (rho = 0.073, *p =* 0.632), neutrophils (rho = −0.097, *p =* 0.523), lymphocytes (rho = −0.117, *p =* 0.440), monocytes (rho = −0.251, *p =* 0.096), eosinophils (rho = −0.121, *p =* 0.427), basophils (rho = −0.199, *p =* 0.196), glucose (rho = −0.079, *p =* 0.609), creatinine (rho = 0.199, *p =* 0.185), sodium (rho = 0.146, *p =* 0.333), potassium (rho = 0.037, *p =* 0.807), chloride (rho = 0.047, *p =* 0.761), calcium (rho = 0.138, *p =* 0.365), phosphorus (rho = 0.185, *p =* 0.236), uric acid (rho = 0.110, *p =* 0.471), transaminase AST (rho = 0.078, *p =* 0.616), transaminase ALT (rho = −0.109, *p =* 0.482), lactate (rho = 0.170, *p =* 0.281), phosphatase (rho = 0.011, *p =* 0.945), or total bilirubin (rho = 0.289, *p =* 0.074) or with the inflammatory markers IL-6 (rho = 0.001, *p =* 0.991), fibrinogen (rho = 0.044, *p =* 0.773), tumor necrosis factor (rho = 0.002, *p =* 0.988), or C-reactive protein (rho = −0.063, *p =* 0.676). No significant effects were observe din the control group.

### 3.3. Nickel Concentration and Its Association with Testosterone and Estradiol

**Figure 4:**
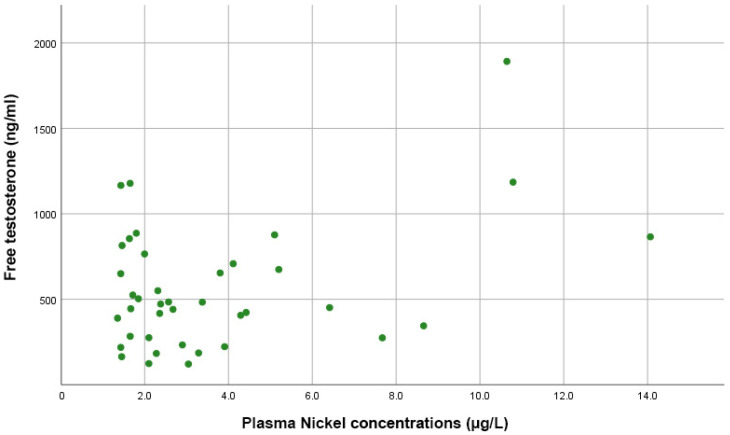
Scatter plot showing the direct proportional correlation between free testosterone levels and plasma nickel levels in PCa patients.

Figure 4 shows that Spearman correlation tests indicated no significant correlations between plasma Ni levels and estradiol (rho = 0.098, *p =* 0.584), total testosterone (rho = −0.055, *p =* 0.729), or free testosterone levels (rho = 0.081, *p =* 0.625).

### 3.4. Plasma Nickel Concentration and Geriatric Evaluation

**Figure 5:**
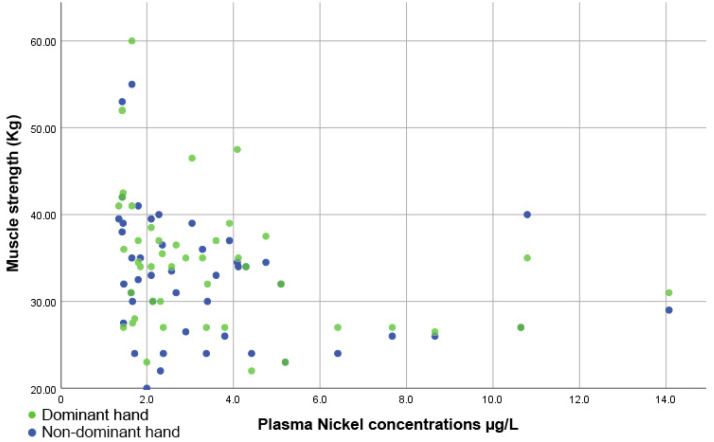
Scatter plot showing the inversely proportional correlation between muscle strength and plasma nickel levels.

Figure 5 indicates that there was an inversely proportional and significant correlation between muscle strength and plasma Ni levels, both for the dominant hand (rho = −0.427, *p =* 0.003) and the non-dominant hand (rho = −0.379, *p =* 0.009).

Finally, there was a significant correlation between the plasma Ni concentration and the Barthel test score (rho = 0.336, *p =* 0.024). However, there was no significant correlation between plasma Ni levels and time spent engaging in physical activity each week (rho = 0.079, *p =* 0.601), sleep quality measured with the AIS (rho = −0.195, *p =* 0.195), cognitive functions assessed with the MMSE (rho = −0.099, *p =* 0.513), or symptoms of depression assessed with the Yesavage scale (rho = −0.067, *p =* 0.661). No significant effects were observed in the control group.

## 4. Discussion

PCa is the most common cancer in men and the second leading cause of cancer deaths among men in Western countries [50]. Multiple factors are thought to be related to an increased risk of PCa, including diet, smoking, lifestyle, and genetic and environmental factors [48,51,52]. The incidence of PCa is highly variable in different countries, suggesting not only an important role for detection practices and treatment availability but also of environmental factors [8,33,53]. Among the latter, exposure to metals like Ni has been postulated as a possible PCa risk factor [8,20].

First, our study revealed a major public health concern, given that many of the patients with and without PCa had high Ni concentrations in their blood. Here, we measured the Ni levels in plasma samples; however, it is difficult to find Ni reference values for plasma in the academic literature, meaning that most of these values are very old. Nonetheless, Høgetveit et al. reported a 10 µg/L limit [54], while Sunderman et al. described maximum Ni levels of 3 µg/L [55], similar to those of 2 µg/L mentioned by Angerer et al. [56]. Normal Ni values of 0.2 µg/L in serum and 1−3 µg/L in urine have also been suggested [57], while older references cite the reference values of 0.05 to 1 µg/L [42]. Finally, the WHO gives a reference value for serum Ni concentrations in healthy individuals without occupational exposure to Ni in the range of 0.14−0.65 µg/L, with the most reliable values being around 0.2 µg/L [58].

Because there is no dose-response relationship for every Ni compound, individual recommendations vary from <10 µg/L [170 nmol/L] to <5 µg/L [85 nmol/L] [59]. The German Research Society regularly publishes a list of maximum concentration in the workplace (MAK) and biological tolerance of working material (BAT) levels. In 2021, the society indicated a BAT reference value of <3 μg/L urine for Ni and its compounds, representing the 95th percentile of its distribution in the general population. However, it did not indicate a value for plasma serum levels [60]. After reviewing eight high-quality studies, Templeton et al. stated that the most reliable reference value for Ni is <0.2 μg/L in serum and <3 μg/L in urine [61]. In our study, we found higher plasma Ni concentrations in PCa patients, although these were not significantly different from the mean levels observed in the control group. These findings differed from previous studies performed in Asian countries, which reported higher blood Ni levels in PCa patients compared to men without PCa or with benign prostatic hyperplasia [21,62].

We also found a correlation between age and Ni concentration in plasma, thereby confirming data reported in younger adults with a mean age of 31 years [63]. This suggested that the Ni burden may be influenced by the duration of environmental exposure. Among the biochemical parameters measured in the blood of the participants, we observed significant associations with renal function parameters (as estimated by the glomerular filtration rate) and plasma urea levels, which may also be related to renal function, among other possibilities. Interestingly, a reduced glomerular filtration rate was inversely associated with plasma Ni levels. Increased serum Ni concentrations have also been reported in patients with chronic renal failure [64,65]. Indeed, the association between plasma Ni levels and renal function parameters was supported by a recent case-control study performed in adult men, in which higher concentrations of Ni compounds in another tissue (i.e., toenails) were observed among patients with renal pathologies compared to healthy controls. The same study found an inverse dose-response relationship between Ni compound concentrations in toenails and renal filtration measured via the glomerular filtration rate index [63]. However, when considering all patients, in the univariate analysis, the glomerular filtration rate did not reach statistical significance, whereas plasma urea concentration remained significant with nickel concentration. However, when we dichotomized patients with reduced (<90 mL/min) or normal (≥90 mL/min) glomerular filtration rate, we found a significantly increased nickel concentration in patients with reduced compared with normal glomerular filtration rate supporting that nickel concentration is increased only in those with renal filtration impairment.

Although it has been known for decades that occupational exposure to Ni has relevant toxic effects, surprisingly little is known about the long-term effects of environmental exposure to Ni on the human kidney or the health status of the general population at low, repeated, or chronic doses. The kidney accumulates excess metal ions, including Ni, through reabsorption, and so it is conceivable that repeated or chronic exposure (as was the case in the elderly participants in this study), even at low levels, may cause altered renal function [66]. Once the kidneys are damaged, increased susceptibility to further insult can accelerate the loss of renal mass and function, which can lead to severe and rapidly progressive diseases such as chronic renal failure [67,68]. In particular, in the case of Ni, the kidney is the main target organ for metal accumulation, and a relationship between Ni exposure and end-stage renal disease has already been described [67,69].

The positive association we identified between Ni levels and plasma urea levels could be related to renal function impairment, as shown in previous work [70]. In contrast, in our work, increased creatinine concentration (as another renal function marker) was not associated with Ni concentration, while other studies reported significant and direct Ni concentration correlations both in blood [63] and urine [71]. One possible explanation for this apparent discrepancy could be due to the fact that most creatinine present in blood is derived from the skeletal muscle amino acids, which may be reduced in older individuals because sarcopenia can lower creatinine values [72,73,74] and thereby mask renal function impairment [75]. In fact, creatinine levels have been shown to be higher in 70-year-old participants than in a comparison population [76], and the blood creatinine level is not a good marker for renal function in older individuals [77].

Regarding the geriatric evaluations we performed, to the best of our knowledge, this study was the first to report to show strong significant and indirect associations between plasma Ni compound levels and muscular strength measured with the handgrip test, thus suggesting a possible link between these two parameters. The possible explanations for these findings are intriguing and may be because of the ability of bivalent Ni ions to compete with calcium signaling in skeletal muscles, given the reports that Ni can block some calcium channels [78,79,80]. In particular, Ni^2+^ blocks T-type voltage-gated Ca^2+^ channels [81,82], as also demonstrated in smooth muscle in preclinical models [82,83].

Thus, this study provides new and compelling evidence that Ni concentrations, even after exposure at low environmental doses, are associated with reduced renal function. However, we must consider the limitations of this research. The number of cases and controls in each study group was relatively small compared to the prevalence of PCA in the general population. The inclusion of patients at all stages in the study is an important limitation since the association with plasma nickel levels could be different in different stages of the disease. Given the nature of the cross-sectional design of this study, further longitudinal studies are warranted in order to infer a possible causal relationship between blood Ni concentration and renal and muscular function impairment.

## Figures and Tables

**Table 1:** Demographic variables.

Variable	PCa	Control	Total
Age: mean	72.24	74.63	
Age: standard error mean	1.380	1.390	
Number of smokers	4	3	7
Educational level:			
No education	9	4	13
Primary	18	26	44
Secondary	12	10	22
University	7	6	13
Employment status:			
Active	3	2	5
Retired	38	43	81
Others	5	1	6
Marital status:			
Married	40	6	46
Widow	2	27	29
Divorced	3	5	8
Others	1	8	9
Totals	46	46	92

## Data Availability

The data presented in this study are available on scientific-based purposes and request from the corresponding author.
